# Protective effect of probiotics on *Salmonella *infectivity assessed with combined *in vitro *gut fermentation-cellular models

**DOI:** 10.1186/1471-2180-11-264

**Published:** 2011-12-15

**Authors:** Annina Zihler, Mélanie Gagnon, Christophe Chassard, Christophe Lacroix

**Affiliations:** 1 Laboratory of Food Biotechnology, Institute of Food, Nutrition and Health, ETH Zürich, Schmelzbergstrasse 7, 8092 Zürich, Switzerland

## Abstract

**Background:**

Accurate assessment of probiotics with targeted anti-*Salmonella *activity requires suitable models accounting for both, microbe-microbe and host-microbe interactions in gut environments. Here we report the combination of two original *in vitro *intestinal models closely mimicking the complex *in vivo *conditions of the large intestine. Effluents from continuous *in vitro *three-stage fermentation colonic models of *Salmonella *Typhimurium infection inoculated with immobilized child microbiota and *Salmonella *were directly applied to confluent mucus-secreting HT29-MTX cell layers. The effects of *Salmonella*, addition of two bacteriocinogenic strains, *Bifidobacterium thermophilum *RBL67 (thermophilicin B67) and *Escherichia coli *L1000 (microcin B17), and inulin were tested on *Salmonella *growth and interactions with epithelial cell layers. *Salmonella *adhesion and invasion were investigated and epithelial integrity assessed by transepithelial electrical resistance (TER) measurements and confocal microscopy observation. Data from complex effluents were compared with pure *Salmonella *cultures.

**Results:**

*Salmonella *in effluents of all reactors of the colonic fermentation model stabilized at mean values of 5.3 ± 0.8 log_10 _cfu/ml effluent. Invasion of cell-associated *Salmonella *was up to 50-fold lower in complex reactor samples compared to pure *Salmonella *cultures. It further depended on environmental factors, with 0.2 ± 0.1% being measured with proximal, 0.6 ± 0.2% with transverse and 1.3 ± 0.7% with distal reactor effluents, accompanied by a similar high decrease of TER across cell monolayers (minus 45%) and disruption of tight junctions. Subsequent addition of *E. coli *L1000 stimulated *Salmonella *growth (6.4 ± 0.6 log_10 _cfu/ml effluent of all 3 reactors) and further decreased TER, but led to 10-fold decreased invasion efficiency when tested with distal reactor samples. In contrast, presence of *B. thermophilum *RBL67 revealed a protective effect on epithelial integrity compared to previous *E. coli *L1000 periods, as reflected by a significant mean increase of TER by 58% in all reactors. Inulin addition enhanced *Salmonella *growth and invasion when tested with distal and proximal reactor samples, respectively, but induced a limited decrease of TER (minus 18%) in all reactors.

**Conclusions:**

Our results highlight the benefits of combining suitable cellular and colonic fermentation models to assess strain-specific first-level host protection properties of probiotics during *Salmonella *infection, providing an efficient system biology tool for preclinical development of new antimicrobials.

## Background

The human colon constitutes a protective and nutrient-rich habitat to trillions of bacteria living in symbiosis with the host [1]. This complex consortium constantly competes with exogenous microbes for attachment sites in the brush border of intestinal epithelial cells, thus preventing pathogens from entering specific ecological niches and gut tissues [2]. Pathogens may however overcome this line of defense, leading to different manifestations of disease. Infectious gastroenteritis caused by non-typhoidal strains of *Salmonella enterica *spp. *enterica *is an important cause of morbidity and mortality worldwide [3]. Due to the increasing incidence of antibiotic resistant and more virulent serovars [4], the use of probiotics with specific anti-*Salmonella *activities is a prevailing interest. Mechanisms by which probiotics inhibit pathogens include competition for nutritional substrates and adhesion sites on intestinal epithelial cells, secretion of antimicrobial substances as well as toxin inactivation and host immunity stimulation [5]. However, *in vivo *mechanistic studies of probiotics and gut microbiota are hindered by ethical considerations, compliance issues and high costs. A variety of *in vitro *gut models have been applied to separately investigate microbe-microbe and simple microbe-host interactions [6-8]. Owing to the complexity of the intestinal environment, suitable models accounting for all intestinal parameters including both the gut microbiota and their substrates and metabolic products as well as the presence of epithelial intestinal cells, represent an indispensable platform for preclinical probiosis assessment.

To investigate the complex gut microbiota *in vitro*, continuous intestinal fermentation models utilizing immobilized fecal microbiota have been developed for the controlled long-term cultivation of gut microbiota with conserved biodiversity [9-11]. Such models allow independent testing of different experimental treatments on both gut microbiota composition and metabolic activity within a single experimental period, using the same microbiota under controlled environmental conditions, which are designed to simulate the proximal, transverse and distal colon of healthy and infected subjects [9-14]. More recently, a three-stage *in vitro *colonic fermentation model of *Salmonella *infection in child colon was used to assess the effects of probiotic and prebiotic treatments on gut microbial behavior and on *S*. Typhimurium infection [15]. The activity of microcin B17-producing *Escherichia coli *L1000 *wt *[16] and bacteriocinogenic *Bifidobacterium thermophilum *RBL67, both exhibiting strong anti-*Salmonella *activity in simple *in vitro *tests [17,18], as well as the microcin B17-negative mutant strain *MccB17-*, were tested in two three-stage models inoculated with the same fecal inoculum. When added to the colonic model, *E. coli *L1000 unexpectedly stimulated *Salmonella *growth in all reactors independently of the microcin B17-phenotype, partly due to a low colonization of the strain in the complex intestinal environment. In contrast, thermophilicin RBL67-producing *Bifidobacterium thermophilum *RBL67 revealed high competitiveness and colonized at high levels but did not reduce *Salmonella *counts, most likely a function of the presence of a very high *Salmonella *population in the *in vitro *model prior to probiotic addition.

Most data available on the mechanistic effects of probiotics on the host are derived from *in vitro *studies with intestinal cells [19]. Such models have also been used to investigate bacterial interactions with the intestinal epithelium during enteric infection [20]. *Salmonella *pathogenesis, for example, has been studied in pure cultures using epithelial Caco-2 and HT-29 cell models [21,22], both of which lack the ability to produce mucus. The mucus-secreting HT29-MTX cell line however, represents more accurate physiological conditions of the gastrointestinal tract for investigating pathogenic behavior during infection, as the presence of mucus has been shown to enhance pathogenicity of pathogens such as *Campylobacter jejuni *[23]. All interaction studies of pathogens and probiotics with intestinal cells have been performed with simple systems of either pure or mixed cultures. Microbe cell interactions are however different when tested in the presence of a complex gut microbiota [24,25]. Gut metabolites such as SCFAs affect epithelial cell metabolism, turnover and apoptosis [26] but may also enhance virulence (e.g. *S*. Typhimurium), by inducing an acid tolerance response or increasing expression of porins [27]. To our knowledge, the effects of an infected gut microbiota, including its metabolites and probiotic treatment on intestinal cells has not been previously reported.

In this study, the mucus-producing HT29-MTX cell model was used to investigate the interaction of *S*. Typhimurium N-15 in presence of a complex intestinal microbiota and to assess the host-protection properties of *E. coli *L1000 and *B. thermophilum *RBL67 sequentially inoculated in the infection model, as well as the protective effect of inulin. Effluent samples were produced in two three-stage continuous colonic models, mimicking the proximal, transverse and distal colon regions and inoculated with immobilized child fecal microbiota and *Salmonella*, and used to test the effects of probiotics and inulin on gut microbiota composition and metabolism, and on *Salmonella *growth [15]. Effluents collected from different fermentation periods were directly applied to HT29-MTX cells to measure *Salmonella *invasion and monitor changes in cellular integrity through both measurement of transepithelial electrical resistance (TER) and confocal microscopy. Data from complex effluents were compared with pure *Salmonella *cultures.

## Results

**Figure 1:**
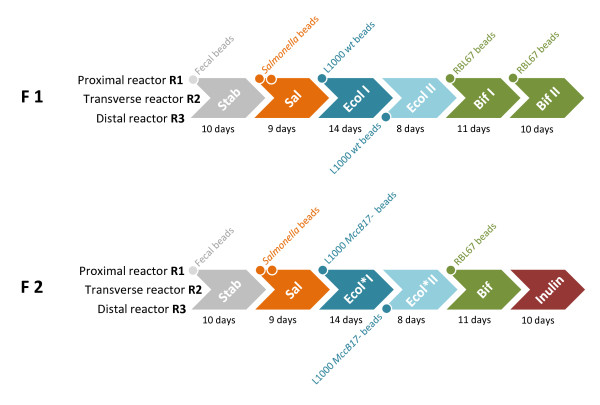
**Experimental design of continuous three-stage colonic fermentations**. Two three-stage continuous fermentation models (F1 and F2) simulating (R1) proximal, (R2) transverse and (R3) distal colonic sections were inoculated with the same immobilized child fecal microbiota, infected with *Salmonella *beads and operated in parallel for a total of 65 days divided into different experimental periods as described previously [15]. For this study, reactor effluents collected during the last 3 days of each experimental period were directly applied onto confluent mucus-secreting HT29-MTX cell layers to detect host-protection properties of different experimental treatments. Data obtained during similar treatments in models F1 and F2 (highlighted in the same color) were not significantly different and therefore used as repetitions: (Stab) initial system stabilization periods, (Sal) *Salmonella *infection periods, (Ecol) *E. coli *L1000 *wt *treatments (microcin B17-producing wild-type strain), (Ecol*) *E. coli *L1000 *MccB17- *treatments (microcin B17-negative mutant strain), (Bif) *B. thermophilum *RBL67 treatments, (Inulin) prebiotic inulin treatment.

**Figure 2:**
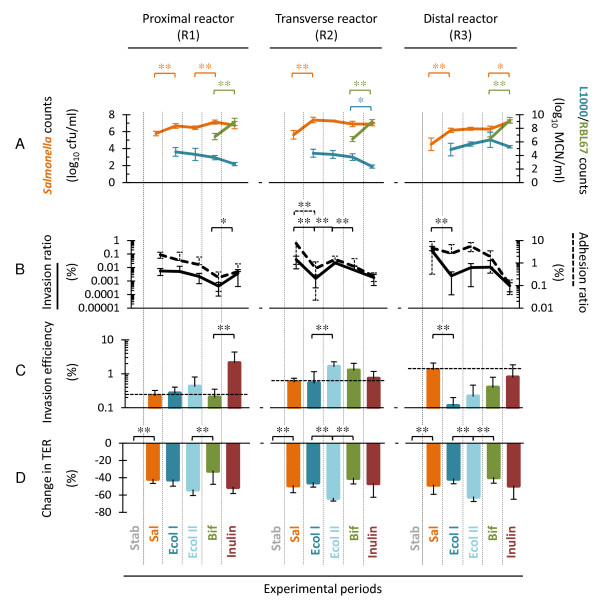
**Bacterial growth, *Salmonella *invasion and TER across HT29-MTX monolayers are affected by experimental and environmental factors**. Values correspond to means ± *SD *(error bars) calculated for effluents of R1, R2 and R3 in F1 and F2 during the last 3 days of each experimental period (Figure 1; *N *= 9 for Bif, *N *= 6 for Stab, Sal and Ecol, *N *= 3 for Inulin). (A) *Salmonella, E. coli *L1000 and *B. thermophilum *RBL67 counts measured by plate counts and real-time qPCR analyses, respectively. Counts of major intestinal bacterial groups were presented previously [15]. (B) Invasion and adhesion ratios, expressed as the percentage of invaded and adhered *Salmonella *related to the total number present in effluents. (C) Efficiency of *Salmonella *to invade HT29-MTX cells, expressed as the percentage of cell-associated *Salmonella*. (D) TER across HT29-MTX cell monolayers measured 1-3 h after incubation with reactor effluents, expressed as ratio to values measured with samples of initial model stabilization periods (Stab). Values reported for subsequent experimental periods and connected with an asterisk are significantly different with the Tukey-Kramer-HSD test (*P < 0.05; **P < 0.01).

**Figure 3:**
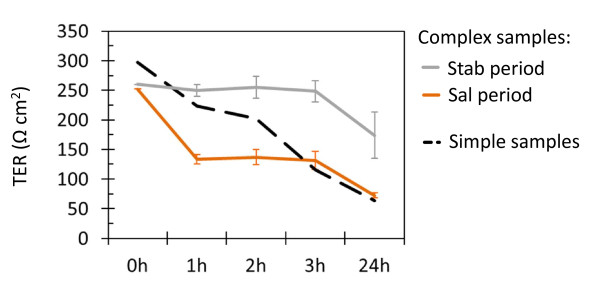
**TER across HT29-MTX monolayers upon application of infected simple and complex fermentation samples**. Values correspond to means ± *SD *(error bars) calculated 1, 2, 3 and 24 h after incubation with complex fermentation effluents of all three reactors from models F1 and F2 obtained during (Stab) initial model stabilization and (Sal) *Salmonella *infection periods (*N *= 6), compared to values measured after incubation with (--x--) *S*. Typhimurium N-15 in DMEM alone.

**Figure 4:**
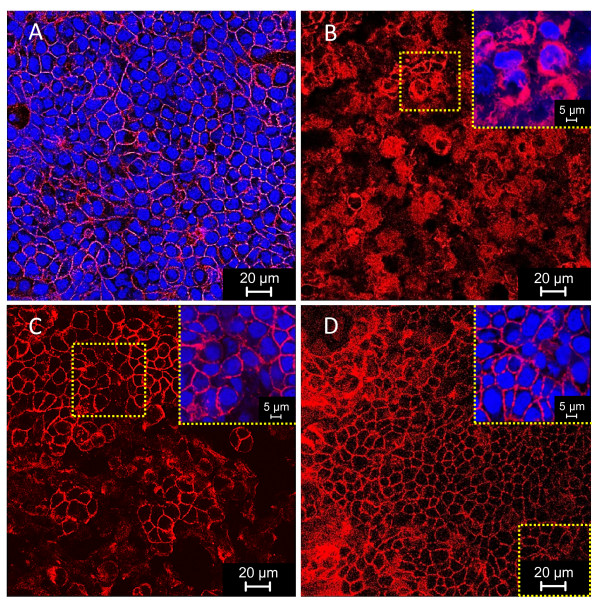
**HT29-MTX monolayer integrity in complex colonic environments is affected by *Salmonella *infection and probiotic treatments**. Tight junctions (in red) and nuclei (in blue) of HT29-MTX cells were stained with phalloidin and DAPI, respectively, after incubation for 90 min with distal reactor effluents of F1 retained at the end of (A, Stab) initial model stabilization, (B, Sal) *Salmonella *infection, (C, Ecol II) *E. coli *L1000 and (D, Bif I) *B. thermophilum *RBL67 periods. Tight junctions were highly disrupted after incubation with effluents from *Salmonella *infection (Sal) compared to initial model stabilization periods (Stab).

Complex reactor effluents were collected during pseudo-steady states (last 3 days) of different experimental periods from two continuous three-stage colonic fermentation models as indicated in Figure 1 and applied directly onto confluent mucus-secreting HT29-MTX cells. Temporal and environmental factors affecting bacterial growth, *Salmonella *invasion and TER across cell monolayers are summarized in Figure 2 and Table 1. TER across cell monolayers after incubation with simple and complex fermentation samples are compared in Figure 3 and the effects on epithelial integrity upon effluent application are shown in Figure 4.

**Table 1:** TER across HT29-MTX monolayers depends on temporal and environmental factors including SCFAs in reactor effluents

		Experimental period					
		
		Stab	Sal	Ecol I	Ecol II	Bif	Inulin
**R1**							

TER	1-3 h	247 ± 24^a^	144 ± 24^bc^	143 ± 22^bc^	114 ± 14^c^	167 ± 34^b^	121 ± 13^bc^
	
	24 h	127 ± 23^a^	69 ± 20^b^	55 ± 11^b^	36 ± 4^b^	130 ± 47^a^	65 ± 14^b^

SCFAs* *(A:P:**B**)*		138 ± 6^a^ *(54:11:**34**)*					179 ± 6^a^ *(44:7:**50**)*

**R2**							

TER	1-3 h	266 ± 19^a^	135 ± 29^b^	144 ± 17^b^	96 ± 4^c^	158 ± 8^b^	142 ± 29^b^
	
	24 h	205 ± 34^a^	74 ± 17^c^	52 ± 4^cd^	34 ± 8^d^	115 ± 19^b^	87 ± 11^bc^

SCFAs* *(A:P:**B**)*		172 ± 6^b^ *(54:14:**32**)*					245 ± 6^b^ *(45:12:**43**)*

**R3**							

TER	1-3 h	240 ± 24^a^	124 ± 30^bc^	141 ± 16^b^	91 ± 6^c^	145 ± 8^b^	121 ± 30^bc^
	
	24 h	190 ± 37^a^	75 ± 17^cd^	77 ± 13^c^	32 ± 11^d^	119 ± 30^b^	91 ± 25^bc^

SCFAs* *(A:P:**B**)*		180 ± 13^b^ *(55:14:**31**)*					234 ± 11^b^ *(46:11:**4**3)*

Mean transepithelial electrical resistance (TER; expressed in Ω cm^2^) ± *SD *were measured after incubation of HT29-MTX cell monolayers for 1-3 h (*N *= 18) and 24 h (*N *= 6) with effluents retained from (R1) proximal, (R2) transverse and (R3) distal colon reactors of F1 and F2 during the last three days of each experimental period. Values with different letters in a row of the same reactor are significantly different according to the Tukey-Kramer-HSD test (*P *< 0.05). *No treatment effects (except for inulin addition) were detected on total short chain fatty acid (SCFA) concentrations (expressed in mM). Mean SCFA concentrations ± *SD *and *(A) acetate: (P) propionate: (B) butyrate *ratios measured during the last three days of non-inulin (*N *= 33) and inulin (*N *= 3) periods are therefore presented. Values with different letters in the same column of different reactors are significantly different with the Tukey-Kramer-HSD test (*P *< 0.05). (Stab) initial system stabilization periods, (Sal) *Salmonella *infection periods, (Ecol) *E. coli *L1000 treatments, (Bif) *B. thermophilum *RBL67 treatments, (Inulin) prebiotic inulin treatment.

### Complex reactor effluents affect TER across HT29-MTX monolayers

*Salmonella *were detected neither in reactor effluents nor after invasion assays in samples obtained at the end of initial model stabilization periods (Stab). Mean TER across HT29-MTX monolayers measured after 1-3 h incubation with effluents from initial model stabilization periods (Stab) were consistent and similar for all reactors (251 ± 23 Ω cm^2^). Furthermore cellular tight junctions were unaffected after 90 min of incubation, as also demonstrated by confocal microscopy for distal reactor effluents of F1 (Figure 4A). 24 h post-incubation, a significant decrease of TER was recorded (Figure 3). A significantly (*P *< 0.05) higher TER was measured with transverse and distal effluents compared to proximal reactor effluents (Table 1), correlating with significantly increased SCFA concentrations in both R2 (177 ± 6 mM) and R3 (187 ± 20 mM) compared to R1 (141 ± 7 mM, Table 1).

### *Salmonella *invasion is a function of environmental factors and affects epithelial integrity

Upon infection of the three-stage continuous fermentation model with *S*. Typhimurium N-15 beads (Sal, Figure 2A), *Salmonella *concentrations in effluents steadily increased and stabilized at significantly (*P *< 0.01) higher levels in proximal (5.8 ± 0.3 log_10 _cfu/ml) and transverse (5.6 ± 0.5 log_10 _cfu/ml) compared to distal colon reactors (4.5 ± 0.7 log_10 _cfu/ml). Invasion efficiency expressed as percentage of cell-associated *Salmonella*, was significantly higher with effluents of R2 (0.6 ± 0.2%; *P *= 0.049) and R3 (1.3 ± 0.7%; *P *= 0.002) compared to R1 (0.2 ± 0.1%) [Sal, Figure 2C]. In contrast, invasion efficiency of pure cultures of *Salmonella *in buffered DMEM was up to 50-fold higher (9.8 ± 2.1%).

Compared to non-infected effluents from initial model stabilization periods (Stab), a large and significant mean decrease of TER across HT29-MTX cell monolayers was measured after 1 h of incubation with effluents of all reactors from *Salmonella *infection periods (Sal, Figure 3). Mean TER values did not differ after 1-3 h of incubation (*P *> 0.05), but significantly decreased after 24 h of incubation (Figure 3). In contrast, TER measured for pure cultures of *S*. Typhimurium N-15 in buffered DMEM showed a continuous and pronounced decrease in TER (Figure 3). Compared to initial model stabilization periods (Stab), mean TER measured 1-3 h after incubation with effluents of all reactors from *Salmonella *infection periods (Sal) were significantly lower (*P *< 0.0001, Table 1), with a mean decrease of 40 ± 4% (Figure 2D). This effect on cell integrity was confirmed by confocal microscopy analysis which demonstrated highly disrupted tight junctions after *Salmonella *infection for distal reactor (R3) effluents of F1 (Figure 4B) compared to initial model stabilization periods (Figure 4A).

### *E. coli *L1000 stimulates *Salmonella *growth yet reduces invasion in the distal colon region

*E. coli *L1000 established itself in the three-stage model at low levels with slightly but non-significantly higher numbers measured in R3 (4.9 ± 0.9 log_10 _MCN/ml) compared to R1 (4.5 ± 0.6 log_10 _MCN/ml) and R2 (4.3 ± 0.6 log_10 _MCN/ml; Figure 2A).

As shown previously [15], the addition of *E. coli *L1000 beads to the intestinal fermentation model enhanced *Salmonella *growth in all colon reactors compared to initial *Salmonella *infection periods (Sal; Figure 2A). However, significantly lower *Salmonella *invasion ratios were measured with transverse and distal reactor effluents (Figure 2B) in comparison with initial *Salmonella *stabilization periods (Sal). Concomitantly, *Salmonella *adhesion ratios remained stable in R3 (Figure 2B), however the efficiency of cell-associated *Salmonella *to invade HT29-MTX cells (Figure 2C) decreased significantly. The second addition of *E. coli *L1000 (Ecol II) had no further effects on *Salmonella *adhesion and invasion ratios in R1 and R3. However, a significantly enhanced (*P *= 0.0004) *Salmonella *invasion ratio was measured with transverse reactor effluents (Figure 2B) compared to the first *E. coli *L1000 period (Ecol I), which was accompanied by a significant increase in invasion efficiency (Figure 2C).

Similar mean TER values were measured with effluents from first *E. coli *L1000 (Ecol I) and *Salmonella *colonization (Sal) periods for all reactors (Table 1, Figure 2D), despite significantly higher *Salmonella *counts (*P *< 0.01) after the addition of *E. coli *L1000 (Figure 2A). TER significantly (*P *> 0.05) decreased by 19% and 26% with transverse and distal reactor effluents respectively (Figure 2D) after the second addition of *E. coli *L1000 (Ecol II) compared to the previous period (Ecol I) while *Salmonella *counts did not change for the two *E. coli *periods (Figure 2A).

### *B. thermophilum *RBL67 exerts a protective effect on epithelial integrity in highly infected environments

*B. thermophilum *RBL67 colonized all reactors of the two three-stage fermentation models, reaching high counts of 6.8 ± 0.5, 6.4 ± 0.4 and 6.5 ± 0.3 log_10 _MCN/ml in R1, R2 and R3, respectively (Bif; Figure 2A). Addition of *B. thermophilum *RBL67 beads increased *Salmonella *counts in R1 compared to the previous *E. coli *L1000 treatment (Ecol II, Figure 2C). However, *Salmonella *invasion efficiency did not change for any of the reactors and the invasion ratio measured with transverse reactor samples significantly decreased during Bif compared to Ecol II periods (Figure 2B).

*B. thermophilum *RBL67 addition (Bif) significantly (*P *= 0.0001) increased the mean TER measured across HT29-MTX cell monolayers applied with effluents of all reactors by 58 ± 17% compared to previous *E. coli *L1000 period (Ecol II, Figure 2D). Mean TER measured after 24 h of incubation with effluents from proximal reactors (130 ± 47 Ω cm^2^) was similar (*P *> 0.05) to initial model stabilization periods (Stab) before *Salmonella *infection (127 ± 23 Ω cm^2^; Table 1). Confocal microscopy analysis revealed high integrity of intracellular junctions upon application of distal colon reactor effluents of F1 after addition of *B. thermophilum *RBL67 (Figure 4D) despite high *Salmonella *counts (6.4 ± 0.6 log_10 _cfu/ml).

### Inulin stimulates *B. thermophilum *RBL67 growth but increases *Salmonella *invasion in proximal colon environments

Addition of inulin induced a significant (*P *= 0.022) increase in *Salmonella *counts (Figure 2A) in R3 compared to previous *B. thermophilum *RBL67 periods (Bif). Furthermore a pronounced enhancement of *B. thermophilum *RBL67 growth (Figure 2A) and an increase in SCFA concentrations and butyrate ratios (Table 1) occurred in all reactors. Inulin supplementation in R1 was accompanied by a significant (*P *= 0.024) increase in the efficiency of *Salmonella *to invade HT29-MTX cells compared to the previous *B. thermophilum *RBL67 period (Bif). This effect was not significant for transverse and distal reactor samples. Inulin treatment also induced a 25%-decrease (*P *= 0.088) in TER after 1-3 h of incubation for effluents of R1 compared to the previous *B. thermophilum *RBL67 periods (Table 1), while a similar but less pronounced tendency was observed for transverse and distal reactors.

## Discussion

Accurate assessment of probiotic-mediated anti-*Salmonella *activities is complicated by the fact that mechanisms involved in enteric protection are the function of many probiotic features. Various interactions take place in complex gut environments, including competition for substrates, direct antagonism by the production of inhibitory substances (e.g. SCFA or bacteriocins), competitive exclusion, and potentially host-mediated effects such as improved barrier function and altered immune response [5,28,29]. It is therefore crucial to consider microbe-microbe as well as host-microbe interactions for the development of probiotics with targeted efficacy. Beyond animal *in vivo *models, combinatorial *in vitro *systems using both gut fermentation and cell models are an integral component in system biology approaches aimed at developing new probiotics [6]. For example, the dynamic TNO-gastrointestinal system (TIM-1) of the human small intestine combined with the Caco-2 cell model was used to investigate the digestive stability and intestinal absorption of lycopene and α-tocopherol [7] Furthermore, adhesion to and cytokine expression of Caco-2 cells was assessed using bacterial cultures, including the probiotic strain *Bifidobacterium longum *DD2004, obtained from a three-stage continuous-culture system (CCS) simulating the proximal and distal large intestine [8]. Results clearly indicate that application of fermentation effluents to intestinal cells represents a valuable platform for assessing epithelial responses as a function of *in vitro *fermentative processes and microbial interactions. In this study, a three-stage continuous intestinal fermentation model closely mimicking conditions in the proximal, transverse and distal colon regions and inoculated with immobilized child feces was used to generate a complex microbiota. For the first time, we report the effects of *Salmonella *in a complex gut microbiota containing metabolites and grown under environmental conditions of the different sections of the colon, on mucus-secreting intestinal HT29-MTX cells. This combined model approach was used to assess host-protecting, anti-*Salmonella *activities of probiotic and prebiotic combinations.

Mean invasion efficiencies of *S*. Typhimurium N-15 into HT29-MTX cells measured in colonic effluents were up to 50-fold lower compared to values measured in simple experimental conditions of a single *Salmonella *strain in DMEM, reflecting different microbe cell interactions in simple systems compared to environments with a complex gut microbiota [24]. Bacterial interactions occurring at the brush-border of HT29-MTX cells may enhance barrier function and diminish *Salmonella *invasion capacity, through the presence of a complex host microbiota, specific metabolites, as well as competition for adhesion sites. SCFAs at physiological concentrations are known to induce a concentration-dependent, reversible change in cellular permeability *in vitro *[25,30]. A higher concentration of total SCFAs in fecal water of adults applied to Caco-2 cells was shown to be associated with an increase in TER in comparison to fecal water obtained from elderly subjects containing lower SCFA concentrations which negatively affected epithelial barrier function [31]. Our results obtained with effluents sampled at the end of model stabilization periods (Stab) were in accordance with these findings. Indeed, a generally higher TER across HT29-MTX cell monolayers was measured after 24 h of incubation for transverse and distal reactor samples with a high concentration of SCFAs accumulating in the *in vitro *model due to the lack of absorption, compared to samples from the proximal reactor. In general, lower TER values were measured during all experimental periods and for all reactors upon effluent exposure for 24 h compared to 1-3 h. As reactor effluents contain a dense and active microbiota, bacterial fermentation and pH reduction can occur during intestinal cell incubation which can negatively affect cell viability thus epithelial integrity [23].

*Salmonella *invasion is influenced by environmental factors such as pH or SCFA concentrations. Upon infection *Salmonella *invasion was generally higher in distal reactors (pH 6.7) compared to proximal (pH 5.7) and transverse (pH 6.2) reactors and inversely related to SCFA concentrations. These results are consistent with findings of Durant *et al*. [32], demonstrating that *Salmonella *entry into HEp-2 cells was higher at pH 7 compared to pH 6 in the presence of 80 mM acetate, 40 mM propionate and 20 mM butyrate. A lower percentage of cell-association and invasion was observed as the concentration of each SCFA increased at pH 6 but not at pH 7 [32]. *Salmonella *invasion into intestinal cells is known to be associated with a rapid disruption of epithelial integrity caused by structural modifications of intercellular junctions that can be assessed by TER measurements [8,33,34]. In this study, we effectively demonstrated that effluents obtained from three-stage *in vitro *colonic fermentation models of *Salmonella *infection and applied directly on confluent and fully differentiated HT29-MTX cells induces a large and significant decrease of TER after 1 h of incubation, compared to non-infected effluents (Figure 3). Visualization of tight junctions by phalloidin staining revealed that intracellular junctions of HT29-MTX cells were not affected by the gut microbiota produced during initial model stabilization (Stab, Figure 4A) but were highly disrupted in the presence of *Salmonella *(Sal, Figure 4B). This is in accordance with results published by Jepson *et al*. [35] where incubation of MDCK monolayers with *S. typhimurium *SL1344 for 60 min was accompanied by a disruption of intracellular junctions.

Addition of *E. coli *L1000 enhanced *Salmonella *growth in all reactors although the efficiency of *Salmonella *in invading HT29-MTX cells significantly decreased in distal reactor (R3) samples. After the addition of *B. thermophilum *RBL67, the invasion efficiency of *Salmonella *decreased most in proximal reactors (R1), despite higher *Salmonella *counts compared to previous Ecol II periods. These results may reflect the influence of environmental requirements for optimal growth of the tested probiotics. *B. thermophilum *RBL67 is acid tolerant and a competitive bacteriocinogenic bacteria [15,18], a trait likely advantageous for competing with other members of the bacterial ecosystem present in proximal colon reactors at pH 5.7. Indeed, *B. thermophilum *RBL67 best colonized and reduced *Salmonella *invasion into HT29-MTX cells at pH 5.7 with proximal reactor samples, while *E. coli *L1000 was more competitive at pH 6.6 in distal colon reactors.

The presence of *E. coli *L1000 in the fermentation model not only enhanced *Salmonella *growth but also induced further disruption of epithelial integrity, a finding which was unexpected. A similar decrease in TER was observed for T84 cells when preventively incubated with *E. coli *Nissle 1917 before addition of *S. dublin *[36]. In contrast, TER values and epithelial integrity after *B. thermophilum *RBL67 addition were significantly enhanced in all reactors of both models although *Salmonella *counts were very high. Several studies reported that live Gram-positive probiotics are able to enhance monolayer barrier function and protect cultured epithelial cells from the effects of infection with invasive pathogens. Preventive treatments with *Lactobacillus acidophilus *and *Streptococcus thermophilus*, for example, were shown to prevent the enteroinvasive *Escherichia coli *(EIEC)-induced decrease in TER of HT29/cl 19A cell monolayers [37]. *Bifidobacterium infantis *and *Bifidobacterium breve *of the probiotic cocktail VSL#3, were shown to improve epithelial integrity of T84 cells and resistance to *Salmonella *invasion [38]. It was suggested that Gram-positive and Gram-negative probiotics use different mechanisms to beneficially modulate the intestinal epithelium and to mediate protection against *Salmonella *[36]. Indeed, the ability of *E. coli *Nissle 1917 and the probiotic mixture VSL#3 to diminish *Salmonella dublin*-induced death of T84 cells was related to the induction of IL-8 secretion by the Gram-negative probiotic, while the Gram-positive probiotic mixture was shown to prevent pathogen-induced decrease in TER and stabilize tight junctions.

Among SCFAs, a special function is assigned to butyrate. In the gut lumen, butyrate is used by epithelial cells as an energy source whereas in tumor cells (e.g. HT29-MTX) butyrate reduces survival by inducing apoptosis and inhibiting proliferation [19,39,40] with concentrations ≥ 8 mM being shown to reduce TER of Caco-2 cells [41]. A similar effect was observed in this study. Inulin induced a strong bifidogenic effect and a shift in SCFA ratios, with a strong increase in butyrate concentrations (Table 1), accompanied by a decrease in TER.

## Conclusions

Our results highlight the benefits of combining suitable cellular and colonic fermentation models to evaluate host protection activity of probiotics during *Salmonella *infection in the presence of commensal gut organisms, providing efficient tools for mechanistic studies *in vitro *which may enhance preclinical development of new antimicrobials. The application of a complex microbiota produced in an *in vitro *fermentation model to HT29-MTX cells revealed that optimal environmental conditions and the impact on *Salmonella *infectivity and intestinal epithelial integrity differed for both probiotic strains tested. *E. coli *L1000 remained at low levels but preferentially colonized the simulated distal colon and also stimulated *Salmonella *growth which was accompanied by a significant disruption of epithelial integrity. In contrast, *B. thermophilum *RBL67 was very competitive and established itself at high levels preferentially in proximal colon reactors. Its presence induced a high increase in TER after 24 h of incubation in all reactors and both models to levels similar to that measured before *Salmonella *addition. Additional studies examining cellular immune responses, including utilizing fecal material from other donors to account for differences in individual gut ecosystems, are necessary in further elucidating the mechanisms of *B. thermophilum *RBL67 and *E. coli *L1000 for treatment of *Salmonella *infections prior to large-scale and costly *in vivo *trials.

## Methods

### Bacterial strains

*Salmonella enterica *spp. *enterica *serovar Typhimurium N-15 (*S*. Typhimurium N-15) was isolated in 2007 from an infected person in Switzerland and obtained from the National Center for Enteropathogenic Bacteria (NENT, Luzern, Switzerland). It was routinely cultivated in tryptic soy broth (TSB, Difco, Basel Switzerland) at 37°C for 18 h.

*E. coli *L1000 *wt*, producing microcin B17 [16], was kindly provided by Hans-Dieter Grimmecke (Laves-Arzneimittel GmbH, Schötz, Switzerland). A mutant strain lacking microcin B17-phenotype (*E. coli *L1000 *MccB17*-) was also used [15]. *B. thermophilum *RBL67, initially isolated from baby feces [42], was obtained from our culture collection.

### Intestinal *in vitro *colonic fermentations

Intestinal colonic fermentations were performed as previously reported [15]. In brief, two three-stage continuous *in vitro *fermentation models (F1 and F2) inoculated with the same immobilized child fecal microbiota were infected with *S*. Typhimurium N-15. These models were operated in parallel for 65 days to test and compare the effects of treatments with probiotic *E. coli *L1000 *wt *and *MccB17*-, followed by *B. thermophilum *RBL67, and prebiotic inulin, on gut microbiota composition, activity, probiotic growth and *Salmonella *colonization [15]. Specific retention times (RT) and pH were applied to the three reactors of each model corresponding to the physiological conditions in child proximal (R1), transverse (R2) and distal (R3) colons: RT = 5 h and pH 5.7 for R1, RT = 10 h and pH 6.2 for R2, and RT = 10 h and pH 6.6 for R3, respectively [43,44].

Continuous fermentations were divided into six consecutive experimental periods illustrated in Figure 1 and presented in detail by Zihler *et al*. [15]. Briefly, the first model F1 used to test *E. coli *L1000 *wt*, included the following conditions: (1) system stabilization [Stab, 10 days], (2) *S*. Typhimurium N-15 beads addition to R1 to induce *Salmonella *infection [Sal, 9 days], (3) first *E. coli *L1000 *wt *beads addition to R1 [Ecol I, 14 days], (4) second *E. coli *L1000 *wt *beads addition to R3 [Ecol II, 8 days], (5) first *B. thermophilum *RBL67 beads addition to R1 [Bif, 11 days], and (6) second *B. thermophilum *RBL67 beads addition to R1 [Bif II, 10 days]. In the second model F2 *E. coli *L1000 *wt *was replaced by *E. coli *L1000 *MccB17- *to assess the effect of microcin B17 phenotype. Similar periods as F1 were tested except for the last period (6) during which prebiotic inulin was tested [Inulin, 10 days].

Effluents (13 ml) were collected daily from each reactor of the two models and processed within 1 h for the enumeration of *S*. Typhimurium N-15 (selective plating), quantification of main bacterial populations (real-time qPCR analyses), and metabolic analysis [15]. Fresh effluents were also directly applied on intestinal HT29-MTX cells.

### Bacterial enumeration

#### Salmonella enumeration by plate counts

*Salmonella *viable cell counts were measured during the last 3 days of each experimental period corresponding to pseudo-steady-state conditions. Effluent samples were serially diluted 10-fold in peptone water (0.1%, pH 7.0) and plated in duplicate on CHROMAgar™*Salmonella *(Becton Dickinson AG, Allschwil, Switzerland). Plates were incubated at 37°C for 48 h.

#### E. coli L1000 and B. thermophilum RBL67 enumeration by real-time qPCR analysis

*E. coli *L1000 and *B. thermophilum *RBL67 concentrations in reactor effluents were estimated by real-time qPCR analysis as described before [15]. Mean copy numbers (MCN/ml) were calculated for the last 3 days of each experimental period of F1 and F2.

### Metabolite analysis

Short-chain fatty acids [SCFA: acetate (A), propionate (P) and butyrate (B)] concentrations in effluent samples were determined in duplicate by high-performance liquid chromatography (HPLC) analysis [12].

### Cell cultures

The human mucus-secreting intestinal colon cancer cell line HT29-MTX [45], obtained after long-term treatment of human carcinoma HT-29 cells with the anti-cancer drug methotrexate [46], was kindly provided by Dr. Thécla Lesuffleur (INSERM, Lille, France). Cells were routinely maintained at 37°C in a humidified incubator (10% CO_2_) in complete Dulbecco's Modified Eagle medium Glutamax (DMEM; Invitrogen AG, Basel, Switzerland) supplemented with 10% (V/V) fetal bovine serum (FBS; Invitrogen AG) and 1% (V/V) antibiotics (10'000 U/ml penicillin + 10'000 μg/ml streptomycin; Invitrogen AG). For invasion assays, cells were seeded in 24-well tissue culture plates (2 cm^2 ^well^-1^; Bioswisstec AG, Schaffhausen, Switzerland) at a concentration of 4 × 10^4 ^cells per well and cultivated for 21 days to reach complete confluence and differentiation. The medium was replaced every 2 days and cell viability was determined by tryptan blue staining (0.1% (V/V) in 10 mM phosphate buffered saline (PBS), pH 7.3). DMEM without antibiotics was used for the last medium change before using the cells for invasion assays.

For transepithelial electrical resistance (TER) measurements, HT29-MTX cells were seeded in cell culture inserts with a 0.45 μm filter membrane and a 0.7 cm^2 ^surface area (24-well culture plate, Millipore AG, Zug, Switzerland) at a concentration of 2.3 × 10^5 ^cells per insert and cultivated as described above.

### Invasion assays

A gentamicin-based assay, as described by Steele-Mortimer *et al*. (2008) but with some modifications, was performed to determine the capacity of *Salmonella *present in reactor effluents to invade HT29-MTX cells. Briefly, 1 ml effluents obtained during the last 3 days of each fermentation period from proximal (R1), transverse (R2) and distal (R3) colon reactors were applied directly in duplicate on cell layers of three consecutive passages and incubated at 37°C for 90 min. To kill non-invading bacteria, cell layers were washed twice with 250 μl PBS before adding 250 μl DMEM supplemented with 150 μg/ml gentamicin (Sigma-Aldrich Chemie GmbH, Buchs, Switzerland) per well followed by an additional incubation period for 60 min at 37°C. After a further washing step with PBS, 250 μl Trypsin-EDTA (1X, Invitrogen) were added followed by another incubation for 10 min. Finally, cells were disrupted by adding 250 μl 0.1% (V/V) Triton X-100 (Sigma) per well and incubating for 10 min before samples were collected for enumeration of invaded *Salmonella*. The same protocol but without gentamicin treatments was used for the determination of cell-associated *Salmonella *(accounting for both invasive and adherent bacteria). The number of adhered *Salmonella *was then calculated from the difference of cell-associated to invaded bacteria. Adhesion and invasion ratios were expressed as the percentage of adhered and invaded bacteria, respectively, related to the total number of *Salmonella *present in effluents. Invasion efficiency measured during different probiotic and prebiotic treatments was expressed as the percentage of invaded bacteria related to the number of cell-associated *Salmonella*.

The same protocol was used to measure the invasion efficiency of *S*. Typhimurium N-15 in pure culture when applied in artificial DMEM medium. Therefore, the pellet of an overnight culture of *Salmonella *obtained by centrifugation (8000 g, 5 min) was diluted in DMEM to reach a concentration of 1.0 × 10^7 ^cfu/ml. 125 μl of this bacterial suspension was added in duplicate to cell monolayers that corresponded to a *Salmonella *concentration (1.3 × 10^6 ^cfu/ml) measured in effluents from the two models during Sal periods.

### Transepithelial electrical resistance (TER) measurements

TER measurements were performed to estimate the degree of cell monolayer's integrity loss that occurs during *Salmonella *infection due to disruption of tight junctions [33]. To measure the epithelial integrity of HT29-MTX cells, 400 μl of effluent was applied directly to the apical compartment of PBS-washed HT29-MTX cell culture inserts that were prepared as previously described. TER measurements were performed before effluent application and after 1, 2, 3 and 24 h of incubation at 37°C. The resistance of cell layers was calculated by subtracting the intrinsic resistance of the filter insert alone from the total measured resistance (filter insert plus cell layer and effluents) and expressed as Ω per cm^2 ^surface area. The same protocol was used to measure the influence of *S*. Typhimurium N-15 on TER of HT29-MTX cells in artificial DMEM medium as presented before.

### Microscopic analysis of tight junctions

To visualize the effects of *Salmonella *infection on cell monolayer integrity before and during probiotic treatments, tight junctions and the nucleus of confluent HT29-MTX cells were fluorescently stained according to previous studies [35,47].

Briefly, HT29-MTX cells were seeded at 9.6 × 10^4 ^cells/ml on a coverslip in a 6-well tissue culture plate and cultured to confluence before incubation with 1 ml of distal colon reactor (R3) effluents from the last day of different treatment periods of F1. DMEM-high glucose without Phenol red (Invitrogen AG, Basel, Switzerland) supplemented with 10% (V/V) fetal bovine serum (FBS; Invitrogen AG) and without antibiotics was used for the last medium change before invasion assays. After incubation of 1 ml effluent for 90 min, cells were washed thrice with PBS and fixed overnight in 1 ml per well of a chilled 4% (V/V) formaldehyde (Sigma-Aldrich Chemie GmbH, Buchs, Switzerland) in PBS solution. After a second washing step (3 times with PBS), cells were permeabilized by treating them with 200 μl of 0.1% Triton X-100 in PBS for 3 min at room temperature. After a third washing step (3 times with PBS), cells were treated with 1 ml of 3% (V/V) albumin bovine serum (BSA, Sigma-Aldrich Chemie GmbH) in PBS to prevent non-specific binding of fluorescent dyes. Tight junctions were stained for 40 min with 1 ml of a 1:200 PBS-diluted stock solution (0.1 mg/ml) of phalloidin-tetramethylrhodamine B isothiocyanate (phalloidin-TRITC, Sigma-Aldrich Chemie GmbH) in methanol, while nuclei were stained for 3 min with 1 ml of a 1:100 PBS-diluted stock solution (5 mg/ml) of 4', 6-diamidino-2-phenylindole (DAPI, Sigma-Aldrich Chemie GmbH) in ultrapure water. After a last washing step, coverslips were mounted inverted on a coverglas by applying one drop of the embedding media Glycergel (DakoCytomation; Glostrup, Denmark). Microscopic analyses were performed with a confocal laser scanning microscope (SP 2, Leica Microsystems, Mannheim, Germany). Different series of images were obtained and stacked by using the Imaris 7 software (Bitplane AG, Zürich, Switzerland).

### Statistical analysis

All statistical analyses were performed using JMP 8.0 for Windows (SAS Institute Inc., Cary, NC, USA). Bacterial counts as well as adhesion and invasion data were log10-transformed to stabilize the variance and normalize residuals values for variance homogeneity.

A one-way analysis of variance (ANOVA) was performed to compare the effects of two consecutive treatments on mean *Salmonella *counts, adhesion and invasion capacities, as well as percentage changes in invasion and adhesion ratios, invasion efficiencies and transepithelial electrical resistance (TER). Measurements during the last 3 days of each fermentation period corresponding to a pseudo-steady-state were used as repetition. *Salmonella *counts, invasion and adhesion ratios, as well as invasion efficiency and TER measured during the last 3 days of each experimental period were not significantly different for F1 and F2, which were inoculated with the same child fecal microbiota immobilized in beads. Therefore, data obtained during system stabilization (Stab), *Salmonella *colonization (Sal) as well as *E. coli *L1000 (Ecol) and *B. thermophilum *RBL67 (Bif) treatment periods of F1 and F2 were used as independent replicates. TER data measured after 1, 2 and 3 h of incubation were not significantly different (*P *> 0.05). Therefore, mean TER values for the three incubation times were reported. Treatment means were compared using the Tukey-Kramer-HSD test with probability levels of *P *< 0.05 and *P *< 0.01.

## Abbreviations

A: Acetate; B: Butyrate; cfu: colony forming units; DMEM: Dulbecco's Modified Eagle medium Glutamax; F1: Continuous three-stage fermentation system 1; F2: Continuous three-stage fermentation system 2; HPLC: High-Performance Liquid Chromatography; MCN: Mean copy numbers; MRS: De Man: Rogosa and Sharpe; P: Propionate; PBS: Phosphate buffered saline; qPCR: Quantitative Polymerase Chain Reaction; R1: Fermentation reactor simulating proximal colon conditions; R2: Fermentation reactor simulating transverse colon conditions; R3: Fermentation reactor simulating distal colon conditions; RT: Retention time; SCFAs: Short-chain fatty acids; TER: Transepithelial electrical resistance; TSB: Tryptic soy broth.

## Authors' contributions

AZ, MG, CC and CL conceived the study. AZ and MG carried out the experiments. AZ, MG, CL and CC analyzed results and drafted the manuscript. All authors read and approved the final manuscript.

## References

[B1] Gaskins HR Croix JA Nakamura N Nava GM Impact of the intestinal microbiota on the development of mucosal defense Clin Infect Dis 2008 46 Suppl 2 S80 S86 discussion S144-S151 18181729 10.1086/523336

[B2] Bernet MF Brassart D Neeser JR Servin AL *Lactobacillus acidophilus *LA 1 binds to cultured human intestinal cell lines and inhibits cell attachment and cell invasion by enterovirulent bacteria Gut 1994 35 483 489 10.1136/gut.35.4.483 8174985 PMC1374796

[B3] Viswanathan VK Hodges K Hecht G Enteric infection meets intestinal function: how bacterial pathogens cause diarrhoea Nat Rev Microbiol 2009 7 110 119 19116615 10.1038/nrmicro2053 PMC3326399

[B4] Claesson MJ O'Sullivan O Wang Q Nikkila J Marchesi JR Smidt H de Vos WM Ross RP O'Toole PW Comparative analysis of pyrosequencing and a phylogenetic microarray for exploring microbial community structures in the human distal intestine PLoS One 2009 4 e6669 10.1371/journal.pone.0006669 19693277 PMC2725325

[B5] Collado MC Isolauri E Salminen S Sanz Y The impact of probiotic on gut health Curr Drug Metab 2009 10 68 78 10.2174/138920009787048437 19149514

[B6] Payne A N Zihler A Chassard C Lacroix C Advances and perspectives in *in vitro *human gut fermentation modeling Trends Biotechnol 2011 doi:10.1016/j.tibtech.2011.06.2011 10.1016/j.tibtech.2011.06.011 21764163

[B7] Deat E Blanquet-Diot S Jarrige JF Denis S Beyssac E Alric M Combining the dynamic TNO-gastrointestinal tract system with a Caco-2 cell culture model: application to the assessment of lycopene and alpha-tocopherol bioavailability from a whole food J Agric Food Chem 2009 57 11314 11320 10.1021/jf902392a 19899761

[B8] Bahrami B Child MW Macfarlane S Macfarlane GT Adherence and cytokine induction in Caco-2 cells by bacterial populations from a three-stage continuous-culture model of the large intestine Appl Environ Microbiol 2011 77 2934 2942 10.1128/AEM.02244-10 21378047 PMC3126424

[B9] Cinquin C Le Blay G Fliss I Lacroix C Immobilization of infant fecal microbiota and utilization in an *in vitro *colonic fermentation model Microb Ecol 2004 48 128 138 10.1007/s00248-003-2022-7 15085302

[B10] Cinquin C Le Blay G Fliss I Lacroix C New three-stage *in vitro *model for infant colonic fermentation with immobilized fecal microbiota FEMS Microbiol Ecol 2006 57 324 336 10.1111/j.1574-6941.2006.00117.x 16867149

[B11] Cinquin C Le Blay G Fliss I Lacroix C Comparative effects of exopolysaccharides from lactic acid bacteria and fructo-oligosaccharides on infant gut microbiota tested in an *in vitro *colonic model with immobilized cells FEMS Microbiol Ecol 2006 57 226 238 10.1111/j.1574-6941.2006.00118.x 16867141

[B12] Cleusix V Lacroix C Vollenweider S Le Blay G Glycerol induces reuterin production and decreases *Escherichia coli *population in an *in vitro *model of colonic fermentation with immobilized human feces FEMS Microbiol Ecol 2008 63 56 64 10.1111/j.1574-6941.2007.00412.x 18028400

[B13] Le Blay G Rytka J Zihler A Lacroix C New *in vitro *colonic fermentation model for *Salmonella *infection in the child gut FEMS Microbiol Ecol 2009 67 198 207 10.1111/j.1574-6941.2008.00625.x 19087202

[B14] Le Blay G Chassard C Baltzer S Lacroix C Set up of a new *in vitro *model to study dietary fructans fermentation in formula-fed babies Br J Nutr 2010 103 403 411 10.1017/S0007114509991796 19751535

[B15] Zihler A Gagnon M Chassard C Hegland A Stevens MJ Braegger CP Lacroix C Unexpected consequences of administering bacteriocinogenic probiotic strains for *Salmonella *populations, revealed by an *in vitro *colonic model of the child gut Microbiology 2010 156 3342 3353 10.1099/mic.0.042036-0 20688827

[B16] Zihler A Le Blay G de Wouters T Lacroix C Braegger CP Lehner A Tischler P Rattei T Hachler H Stephan R *In vitro *inhibition activity of different bacteriocin-producing *Escherichia coli *against *Salmonella *strains isolated from clinical cases Lett Appl Microbiol 2009 49 31 38 10.1111/j.1472-765X.2009.02614.x 19413755

[B17] von Ah U Identification of *Bifidobacterium thermophilum *RBL67 isolated from baby faeces and partial purification of its bacteriocin PhD thesis 2006 Diss Nr 16927, Swiss Federal Institute of Technology Zurich (ETHZ), Zurich, Switzerland

[B18] von Ah U Mozzetti V Lacroix C Kheadr EE Fliss I Meile L Classification of a moderately oxygen-tolerant isolate from baby faeces as *Bifidobacterium thermophilum* BMC Microbiol 2007 7 79 10.1186/1471-2180-7-79 17711586 PMC2045100

[B19] Mennigen R Bruewer M Effect of probiotics on intestinal barrier function Ann N Y Acad Sci 2009 1165 183 189 10.1111/j.1749-6632.2009.04059.x 19538305

[B20] Gagnon M Zihler A Chassard C Lacroix C Malago JJ, Koninkx JFJG, Marinsek-Logar R Ecology of probiotics and enteric protection Probiotic Bacteria and Enteric Infections-Cytoprotection by probiotic bacteria 2011 1 Springer Science + Business Media B.V. 65 85

[B21] Weinstein DL O'Neill BL Hone DM Metcalf ES Differential early interactions between *Salmonella enterica *serovar Typhi and two other pathogenic *Salmonella *serovars with intestinal epithelial cells Infect Immun 1998 66 2310 2318 9573122 10.1128/iai.66.5.2310-2318.1998 PMC108196

[B22] Rabot S Rafter J Rijkers GT Watzl B Antoine JM Guidance for substantiating the evidence for beneficial effects of probiotics: impact of probiotics on digestive system metabolism J Nutr 2010 140 677S 689S 10.3945/jn.109.113738 20107147

[B23] Alemka A Clyne M Shanahan F Tompkins T Corcionivoschi N Bourke B Probiotic colonization of the adherent mucus layer of HT29MTXE12 cells attenuates *Campylobacter jejuni *virulence properties Infect Immun 2010 78 2812 2822 10.1128/IAI.01249-09 PMC2876579 20308300

[B24] Cencic A Langerholc T Functional cell models of the gut and their applications in food microbiology--a review Int J Food Microbiol 2010 141 Suppl 1 S4 S14 20444515 10.1016/j.ijfoodmicro.2010.03.026 PMC7173225

[B25] Bahrami B Macfarlane S Macfarlane GT Induction of cytokine formation by human intestinal bacteria in gut epithelial cell lines J Appl Microbiol 2011 110 353 363 10.1111/j.1365-2672.2010.04889.x 21070518

[B26] Stoidis CN Misiakos EP Patapis P Fotiadis CI Spyropoulos BG Potential benefits of pro- and prebiotics on intestinal mucosal immunity and intestinal barrier in short bowel syndrome Nutr Res Rev 2010 1 9 10.1017/S0954422410000260 20961485

[B27] Rishi P Pathak S Ricke SC Short chain fatty acids influence virulence properties of *Salmonella enterica *serovar Typhimurium J Environ Sci Health B 2005 40 645 657 10.1081/PFC-200061576 16047886

[B28] O'Toole PW Cooney JC Probiotic bacteria influence the composition and function of the intestinal microbiota Interdiscip Perspect Infect Dis 2008 2008 175285 19277099 10.1155/2008/175285 PMC2648622

[B29] Corr SC Hill C Gahan CG Chapter 1 Understanding the mechanisms by which probiotics inhibit gastrointestinal pathogens Adv Food Nutr Res 2009 56 1 15 19389605 10.1016/S1043-4526(08)00601-3

[B30] Kalliomaki M Antoine JM Herz U Rijkers GT Wells JM Mercenier A Guidance for substantiating the evidence for beneficial effects of probiotics: prevention and management of allergic diseases by probiotics J Nutr 2010 140 713S 721S 10.3945/jn.109.113761 20130079

[B31] Gill CI Heavey P McConville E Bradbury I Fassler C Mueller S Cresci A Dore J Norin E Rowland I Effect of fecal water on an *in vitro *model of colonic mucosal barrier function Nutr Cancer 2007 57 59 65 10.1080/01635580701268220 17516863

[B32] Durant JA Lowry VK Nisbet DJ Stanker LH Corrier DE Ricke SC Short-chain fatty acids affect cell-association and invasion of HEp-2 cells by *Salmonella typhimurium* J Environ Sci Health B 1999 34 1083 1099 10.1080/03601239909373246 10565427

[B33] Sekelja M Berget I Naes T Rudi K Unveiling an abundant core microbiota in the human adult colon by a phylogroup-independent searching approach ISME J 2011 5 519 531 10.1038/ismej.2010.129 20740026 PMC3105728

[B34] Alemka A Clyne M Shanahan F Tompkins T Corcionivoschi N Bourke B Probiotic colonization of the adherent mucus layer of HT29MTXE12 cells attenuates *Campylobacter jejuni *virulence properties Infect Immun 2010 78 2812 2822 10.1128/IAI.01249-09 20308300 PMC2876579

[B35] Jepson MA Collares-Buzato CB Clark MA Hirst BH Simmons NL Rapid disruption of epithelial barrier function by *Salmonella typhimurium *is associated with structural modification of intercellular junctions Infect Immun 1995 63 356 359 7806378 10.1128/iai.63.1.356-359.1995 PMC173001

[B36] Otte JM Podolsky DK Functional modulation of enterocytes by gram-positive and gram-negative microorganisms Am J Physiol Gastrointest Liver Physiol 2004 286 G613 G626 10.1152/ajpgi.00341.2003 15010363

[B37] Resta-Lenert S Barrett KE Live probiotics protect intestinal epithelial cells from the effects of infection with enteroinvasive *Escherichia coli *(EIEC) Gut 2003 52 988 997 10.1136/gut.52.7.988 12801956 PMC1773702

[B38] Madsen K Cornish A Soper P McKaigney C Jijon H Yachimec C Doyle J Jewell L De Simone C Probiotic bacteria enhance murine and human intestinal epithelial barrier function Gastroenterology 2001 121 580 591 10.1053/gast.2001.27224 11522742

[B39] de Los Reyes-Gavilan CG Suarez A Fernandez-Garcia M Margolles A Gueimonde M Ruas-Madiedo P Adhesion of bile-adapted *Bifidobacterium *strains to the HT29-MTX cell line is modified after sequential gastrointestinal challenge simulated *in vitro *using human gastric and duodenal juices Res Microbiol 2011 162 514 519 10.1016/j.resmic.2011.03.009 21419219

[B40] Mirold S Ehrbar K Weissmuller A Prager R Tschape H Russmann H Hardt WD *Salmonella *host cell invasion emerged by acquisition of a mosaic of separate genetic elements, including *Salmonella *pathogenicity island 1 (SPI1), SPI5, and sopE2 J Bacteriol 2001 183 2348 2358 10.1128/JB.183.7.2348-2358.2001 11244077 PMC95144

[B41] Peng L He Z Chen W Holzman IR Lin J Effects of butyrate on intestinal barrier function in a Caco-2 cell monolayer model of intestinal barrier Pediatr Res 2007 61 37 41 10.1203/01.pdr.0000250014.92242.f3 17211138

[B42] Touré R Kheadr E Lacroix C Moroni O Fliss I Production of antibacterial substances by bifidobacterial isolates from infant stool active against *Listeria monocytogenes* J Appl Microbiol 2003 95 1058 1069 10.1046/j.1365-2672.2003.02085.x 14633035

[B43] Fallingborg J Christensen LA Ingeman-Nielsen M Jacobsen BA Abildgaard K Rasmussen HH Rasmussen SN Measurement of gastrointestinal pH and regional transit times in normal children J Pediatr Gastroenterol Nutr 1990 11 211 214 10.1097/00005176-199008000-00010 2395061

[B44] Wagener S Shankar KR Turnock RR Lamont GL Baillie CT Colonic transit time--what is normal? J Pediatr Surg 2004 39 166 169 discussion 166-169 10.1016/j.jpedsurg.2003.10.002 14966733

[B45] Lesuffleur T Barbat A Dussaulx E Zweibaum A Growth adaptation to methotrexate of HT-29 human colon carcinoma cells is associated with their ability to differentiate into columnar absorptive and mucus-secreting cells Cancer Res 1990 50 6334 6343 2205381

[B46] Van de Wiele TR Verstraete W Siciliano SD Polycyclic aromatic hydrocarbon release from a soil matrix in the *in vitro *gastrointestinal tract J Environ Qual 2004 33 1343 1353 10.2134/jeq2004.1343 15254116

[B47] Kim KP Loessner MJ *Enterobacter sakazakii *invasion in human intestinal Caco-2 cells requires the host cell cytoskeleton and is enhanced by disruption of tight junction Infect Immun 2008 76 562 570 10.1128/IAI.00937-07 18070906 PMC2223463

